# AQVx—An Interactive Visual Display System for Air Pollution and Public Health

**DOI:** 10.3389/fpubh.2020.00085

**Published:** 2020-03-24

**Authors:** Grant J. Williamson, Christopher Lucani

**Affiliations:** School of Natural Sciences, University of Tasmania, Hobart, TAS, Australia

**Keywords:** smoke, wildfire, prescribed, visualization, transport, radar, exposure

## Abstract

Fine particulate matter emissions (PM_2.5_) from landscape biomass fires, both prescribed and wild, pose a significant public health risk, with smoke exposure seasonally impacting human populations through both highly concentrated local plumes, and more dispersed regional haze. A range of technologies now exist for mapping and modeling atmospheric particulate concentration, including low-cost mobile monitors, dispersion and chemical transport modeling, multi-spectral earth observation satellites, weather radar, as well as publicly available real-time data feeds from agencies providing information about fire activity on the ground. Ubiquitous smart phone availability also allows instant public reporting of both health symptoms and smoke exposure. We describe a web-based visual display interface, Air Quality Visualization (AQVx), developed to allow the overlaying, synchronization and comparison of a range of maps and data layers, in order to both assess the potential public health impact of landscape fire smoke plumes, and the accuracy of dispersion models. The system was trialed in the state of Victoria, in south-eastern Australia, within the domain of the AQFx chemical transport model, where large-scale annual prescribed burning operations (~11,000 km^2^ yr) are carried out, and where extreme wildfires frequently occur during the summer months. AQVx, coupled with the ARSmoke smart phone application, allowed managers to rapidly validate modeled smoke transport against satellite imagery, and identify potential exposure risks to populated areas.

## 1. Introduction

Smoke emissions from landscape fires are primarily composed of fine particulate matter (PM), with the fraction with an aerodynamic diameter <2.5 μm (PM_2.5_) increasingly recognized as having a significant human health impact (1). Smoke from landscape fires can elevate PM concentrations well above background levels over large areas, often a long distance from the emission source due to wind transport of the plume. Landscape fire smoke pollution episodes can be brief and relatively local but with extreme exposure concentrations (2), or produce longer-lasting regional haze, as is often observed as a result of burning in south-east Asia (3). Biomass smoke has been considered in the past to have similar health effects to urban air pollution (4), but recent studies that attempt to discriminate the specific effects of wildfire smoke have found associations between wildfire smoke exposure and respiratory health, including exacerbation of asthma and chronic obstructionary pulmonary disease (COPD) (1). In Australia, studies focusing on wildfire smoke exposure in Darwin (5), Brisbane (6), the Sydney region (7, 8), Victoria (9), and Tasmania (10) have identified increases in mortality, respiratory health effects and at times cardiovascular impacts.

In the state of Victoria, in south-eastern Australia, community and fire management focus has been placed on prescribed burning programs to reduce wildfire risk. Following the devastating “Black Saturday” fires in February 2009, which resulted in 173 fatalities and burned over 450,000 ha (11), the 2009 Victorian Bushfires Royal Commission (12) recommended an annual rolling 5% statewide prescribed burning target in order to manage forest fuel mass and reduce wildfire risk. In 2015, this policy was reviewed (13) and a shift toward targeted risk reduction was implemented, with the recognition that a flat 5% area target was not achievable or sustainable, and could result in excessive prescribed burning application in areas that would make little improvement to public risk or ecological values. The approach that has been adopted implements fire spread and scenario modeling in order to determine optimal areas to be burnt in order to minimize residual wildfire risk (14). This has resulted in a significant reduction in total annual area burnt by prescribed fire, from a peak of 281,950 ha in the 2012–2013 season to just 75,857 ha in the 2017–2018 season (15), but given the burn program is targeted toward reducing risk to communities and assets, fires and the resulting smoke plumes are likely to more frequently impact populated areas due to their proximity.

In order to assist in prescribed burn planning and identification of potential smoke plume impacts on populated areas, a chemical transport and dispersion model is run daily to project smoke transport from wild and prescribed fires, and the interaction of smoke with other emissions sources. Atmospheric dispersion models (16) can provide high resolution forecasts of plume location, transport, and PM concentrations, but require accurate meteorological and emissions parameterization, and there is a need to validate them against observations to ensure their accuracy and utility (17). On-ground PM monitors provide the best means of validation, as they provide accurate measurements at precise point locations with an observation frequency compatible with that of dispersion model output. However, historically PM monitors have primarily been established to monitor urban, traffic and industrial pollutants in major population centers, and have poor coverage in rural, sparsely-populated areas where landscape fires generally occur (18). Therefore, there is scope to utilize other means of detecting smoke plumes and measuring PM concentrations in validating atmospheric dispersion model output.

Earth observation satellite platforms that provide detections of smoke, fire and aerosol/PM concentration are becoming increasingly available. Geostationary satellites, such as the Japan Meteorological Agency's Himawari-8, deliver extremely frequent imagery in a range of spectral bands, and can provide valuable insight into fire location and smoke transport in close to real time. Sun-synchronous satellites, such as NASA's Aqua and Terra satellites, which carry the Moderate Resolution Imaging Spectroradiometer instrument (MODIS) and the Suomi NPP satellite with the Visible Infrared Imaging Radiometer Suite (VIIRS) provide coverage multiple times a day, and allow detection of active fire hotspots in near real-time, as well as estimations of aerosol optical depth (AOD), a proxy for atmospheric column particulate concentration. The European Space Agency's Sentinel-2 satellites provide high-resolution (10–30 m ground pixel) coverage approximately every 5 days, providing detailed imagery of fire burnt areas. These satellite observations provide a range of measurements and imagery useful for validating both dispersion and transport model inputs, such as fire location and intensity, and model outputs, such as plume location, dispersion direction, and PM concentration. A visual interface that allows integrated access and overlaying of the latest satellite imagery and model output, over multiple time steps, can enable a qualitative and quantitative comparison of model output with measurements and observations.

## 2. Methods and Data

**Figure 1:**
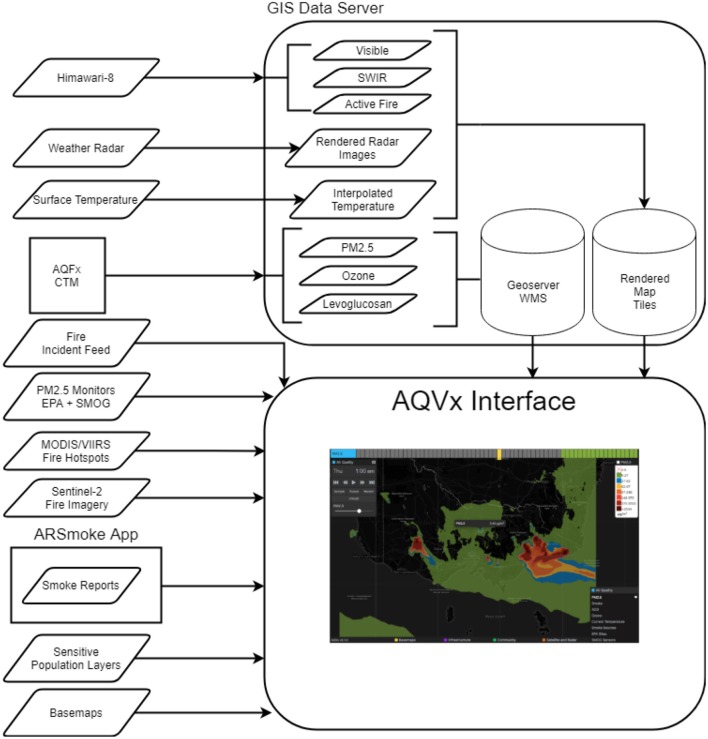
The AQVx system integrates chemical transport modeling with on-the-ground air quality sensors, satellite imagery, radar, crowd-sourced reports of smoke and health symptoms, fire-related incidents and planned burns and community infrastructure.

AQVx (Air Quality Visualization) is a web-based interactive visual analysis system that integrates chemical transport dispersion modeling (19) of PM_2.5_, ozone, and levoglucosan with on-the-ground air quality sensors, crowd-sourced reports of smoke and health symptoms, satellite imagery, radar, fire-related incidents and planned burns and infrastructure (Figure 1). Trialed during the 2019 annual prescribed burning operations in Victoria, the AQVx system allowed fire managers from the Department of Environment, Land, Water and Planning (DELWP) to query and validate modeled air quality, and identify potential exposure risks to populated areas.

### 2.1. Data Sources

The AQVx system ingests, processes and displays a range of model, satellite data sources, listed in Table 1. Data are acquired from these sources automatically at appropriate intervals, for example CSIRO Chemical Transport Model output is transferred once every 24 h when modeling is complete, while Himawari-8 satellite data is transferred every 10 min. System administrators are notified if data acquisition and ingestion failed for any reason so end users can be notified, and missing time steps for layers are not displayed on the interactive timeline.

**Table 1:** Air quality, satellite, and radar data sources included in AQVx.

**Layer type**	**Layer**	**Description**	**Total time period**	**Time segment**	**Spatial resolution**	Source*
Air Quality	PM_2.5_	CSIRO CTM PM_2.5_	144 h past, 24 h forecast	1 h	3 km smoothed	CSIRO, BoM
	Smoke	CSIRO CTM levoglucosan scaled to PM_2.5_	144 h past, 24 h forecast	1 h	3 km smoothed	CSIRO, BoM
	Ozone	CSIRO CTM ozone	144 h past, 24 h forecast	1 h	3 km smoothed	CSIRO, BoM
	Current temperature	Interpolated temperature	Current	n/a	1 km smoothed	AirRater/BoM
	Smoke sources	Planned burns and smoke-related incidents	24 h	1 h	n/a	EMV
	EPA sites	Daily and hourly PM_2.5_ for available EPA sites/stations	24 h	1 h	n/a	EPA
	SMOG sensors	Daily and hourly PM_2.5_ recorded from SMOG sensors	24 h	1 h	n/a	CSIRO
Satellite and radar	Radar Melbourne	128 km Melbourne radar	2 h	6 min	Variable	BoM
	Radar Yarrawonga	128 km Yarrawonga radar	1.5 h	30 min	Variable	BoM
	Himawari IR	Himawari 0.86 μm band	18 h	10 min	1 km	BoM, JMA
	Himawari active fires	Thermal infrared detections of active fires	18 h	10 min	1 km	CSIRO, BoM, JMA
	Himawari Vis.	Himawari true-color visible spectrum	18 h	10 min	500 m	BoM, JMA
	Sentinel-2 SWIR	Short-wave infrared composite based on bands 21.9, 0.865, 0.665	4 weeks	Daily	20 m	S-HUB, ESA
	Geoscience hotspots	Recent detections of active fire hotspot locations from MODIS and VIIRS platforms	72 h	–	–	GEO-AU

#### 2.1.1. CSIRO Chemical Transport Model

**Figure 2:**
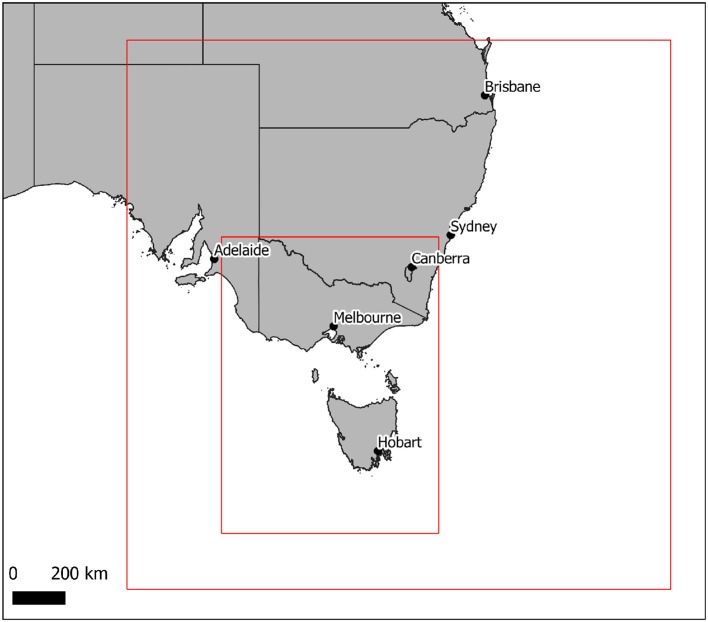
Two domains of the AQFx chemical transport model, with the south-eastern Australia domain run at a 9 km resolution, and the inner Victoria-Tasmania domain run at a 3 km resolution.

The CSIRO Chemical Transport Model (19) (CTM) was implemented to be run daily in nested domains, with an outer domain covering the Australian region at 30 km resolution, a domain over south-eastern Australia with a 9 km resolution, and an inner domain covering the states of Victoria and Tasmania at a 3km resolution (Figure 2). This implementation of the transport model is termed AQFx (Air Quality Forecast), and is forced with meteorology from the Australian Bureau of Meteorology's ACCESS numerical forecast model (ACCESS-R for outer domains, ACCESS-VT for inner domains), and incorporates emission inventories from urban, traffic, industrial, biogenic and sea-salt production. Landscape fire emissions for Victoria are generated from Phoneix fire behavior model simulations (20) using fire area polygons provided by DELWP, and outside Victoria satellite fire hotspots are provided by Geoscience Australia's Sentinel service (21), which incorporates hotspot detections by the MODIS and VIIRS instruments. Outer-domain generic particle concentrations are generated from GLOMAP simulations (22). Model output is available at 10:00 AEST each day, and is automatically downloaded to the AQVx mapping server. Model output NetCDF files are processed using GDAL v2.2.2 (23) and R v3.4.4 (24), integrated into a 7-days archive, and transferred to GeoServer v2.12.1 (25) for rendering and display though a standard web map service (WMS).

#### 2.1.2. Satellite Imagery

The Himawari-8 satellite (26) launched by the Japan Meteorological Agency became operational in July 2015, in a geostationary orbit at 140.7° east, that provides coverage of Australia. The Advanced Himawari Imager (AHI) instrument onboard Himawari-8 returns imagery in 18 bands, with a ground resolution ranging from 0.5 to 2 km, and a temporal resolution of 10 min, or 2.5 min for defined target areas. Every 10 min, a set of Himawari-8 bands are downloaded automatically from the Australian Bureau of Meteorology, comprising visible red (0.64 μm), green (0.51 μm), and blue (0.47 μm), near infrared (0.86, 1.61, 2.26 μm), and thermal infrared (3.89 μm). Red, green, and blue channels are combined to form a true-color visible image, the 0.86 μm band provides an monochromatic image for night-time. Static map tiles of satellite imagery are rendered using GDAL v2.2.2 (23) and served by a web server.

Short-wave infrared composite based on bands 21.9, 0.865, and 0.665 μm from the European Space Agency's Sentinel-2 multispectral satellite (27) were integrated from Sentinel Hub's public WMS server and integrated directly into the AQVx interface without pre-processing on the AQVx server.

#### 2.1.3. Radar

Radar files in PPI format were downloaded every 6–30 min, depending on radar temporal resolution, from the Bureau of Meteorology for the Melbourne and Yarrawonga (northern Victoria) weather radar sites. While typically used to visualize precipitation, weather radar can detect returns from ash particles in smoke plumes from large fires, providing an additional source of plume verification. Radar files were interpreted in R v3.4.4 (24), converted to projected cartesian coordinates, and rendered to map tiles for display.

#### 2.1.4. Active Fire Hotspots

Active fire hotspots were provided by Geoscience Australia's Sentinel service (Geoscience Australia 2019), which incorporates hotspot detections by the MODIS and VIIRS instruments. Real-time active fire visualization based on thermal infrared wavelengths was generated using a combination of the 1.61, 2.26, and 3.89 μm bands from Himawari-8 combined according to a method developed by Naomi Benger (Australian Bureau of Meteorology, personal communication).

#### 2.1.5. Smoke Sources

Every 10 min the AQVx server processed the VicEmergency feed of alerts and prescribed burns and filters for smoke-related alerts and burns.

#### 2.1.6. Air Quality

Twenty-four and 1-h PM_2.5_ averages from low-cost particulate sensors deployed in regional Victoria, and 8- and 1-h PM_2.5_ and air quality index (AQI) from EPA monitors via EPA's Air Quality API web service, were incorporated into the database for display at point locations.

#### 2.1.7. Infrastructure and Population

A list of government and non-government primary and secondary schools for Victoria was sourced from the Victorian Department of Education and Training, as well as aged care facilities derived from a Department of Health list of aged care services subsidized by the Australian Government under the Aged Care Act 1997. Point locations of these facilities were displayed in the AQVx system to help identify sensitive populations in the path of modeled smoke plumes. To enable visualization of broader population exposure, AQVx integrated Geoscience Australia's National Exposure Information System (NEXIS) population density product, providing the number of people per 10 km^2^.

#### 2.1.8. Community

The smartphone app, *AirRater Smoke*, was developed alongside AQVx to crowd-source smoke and symptoms reports from users. The *Airrater Smoke* application does not show the same information as the AQVx interface, but instead provides a simpler map of monitor-derived particulate concentrations and other meteorological data, as well as providing an interface for submitting symptom and smoke reports. Users submitted symptom reports when experiencing symptoms, such as shortness of breath or eye irritation. The collection of symptom data is not intended for epidemiological analysis, as user submission of symptoms may be biased, with users more likely to use the app and submit symptoms when smoke is visible and present. Rather, it is intended as a rapid health surveillance tool, so operators can immediately identify locations where smoke-related symptoms are being reported, and determine whether particulate concentrations, according to ether model or monitor data, are elevated where symptoms are being reported. This provides an additional validation of model output.

Users also submitted smoke reports when they could see and/or smell smoke in the distance or at their current location. If the smoke was in the distance the user was asked to take a photograph with their phone, and this image was stored on the AQVx server along with the orientation of the device in order enable triangulation of the plume location from multiple reports. Plume triangulation was performed by generating a raster image over the area of interest, with each cell's value calculated as the sum of a function of the distance of the cell to the estimated “focal location” of each selected smoke report, based on camera orientation. Cells >15° in angular distance from the camera orientation vector, or further than 20 km from the camera location, are given a value of zero, and cell values increase as they approach the orientation vector and camera location. Cells with higher total values represent areas where a smoke plume was more likely to be present.

### 2.2. AQVx Interface

The AQVx interface was developed as a web-based tool to maximize accessibility to operators and to facilitate immediate deployment of fixes and updates. The tool was made available through a password-protected web address.

**Figure 3:**
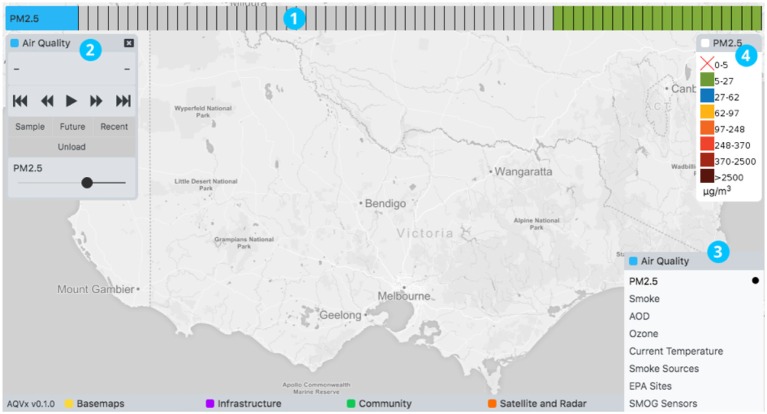
The AQVx interface showing four key elements: (1) a time track for each layer that has time series data available; (2) layer controls provide layer-specific options and controls; themed menus (3) provide access to map layers; a legend is displayed for relevant layers (4).

Figure 3 provides an overview of the AQVx interface features. The tool is map-centric, with data visually represented as map layers of map tiles or map markers. Map layers were accessed through menus available at the bottom of the tool, grouped in four broad themes: Air Quality, Satellite and Radar, Community, and Infrastructure. Layer controls provide layer-specific functionality and settings, such as layer opacity. A legend is displayed for all relevant map layers. Raw PM_2.5_ concentrations as μg m^−3^ are provided for the monitor and model layers, rather than a unitless Air Quality Index (AQI), because the Australian Ambient Air Quality National Environment Protection Measure (NEPM) monitors regulatory exceedences in these units. In addition, the AQVx system is intended specifically for monitoring pollution relevant to landscape fire, and neither the AQFx model, nor individual monitoring stations, measure the complete suite of pollutants that comprise the AQI.

Where time series data is available for a map layer it is made available through an innovative “time track” feature that appeared at the top of the tool. Time tracks provide flexibility in providing access to time series data from feeds that may have significantly different total time periods and time steps. For example, radar may be available for a total time period of 2 h with 6-min time steps, while CTM output provides hourly time steps for a total of 144 h.

**Figure 4:**
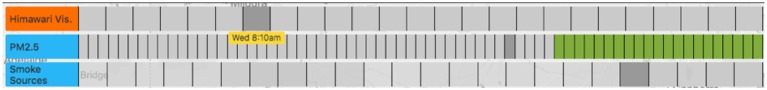
The time track provides a simple and intuitive mechanism for synchronizing and displaying time-series data from different sources while maintaining a simple representation of the data available. The entire time track represents the total duration available with each block representing a single time step. Hovering over a time step displays the time and blocks in other time tracks with coincident times are highlighted.

Within a time track, time series are divided into blocks that represent individual time steps (Figure 4). Hovering the mouse pointer over a block displays the time the block represents. When multiple layers and time tracks are loaded, hovering over a block in one time track automatically highlights blocks in other time tracks with coincident time. This provides a simple and intuitive mechanism for synchronizing and displaying data from different sources while maintaining a simple representation of the data available. For a dynamic perspective, time series data can be animated using layer animation controls.

**Figure 5:**
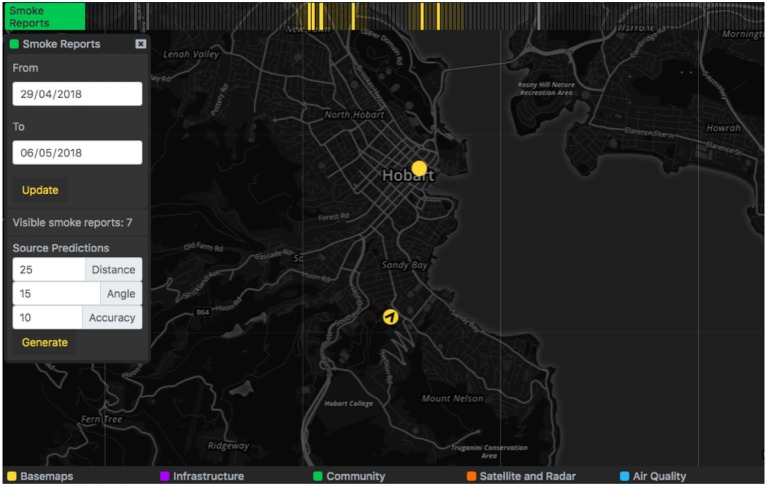
Map markers indicate smoke reports submitted via the AirRater Smoke app. The time track groups reports by the hour.

**Figure 6:**
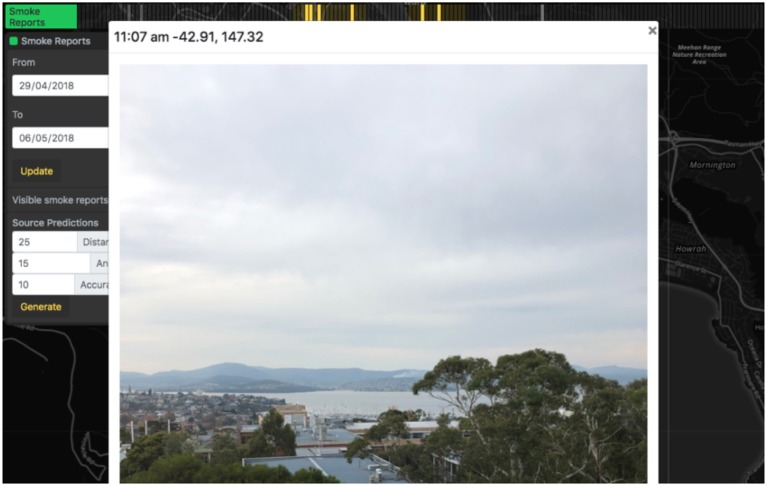
User-submitted photos of smoke are accessible through the AQVx interface.

**Figure 7:**
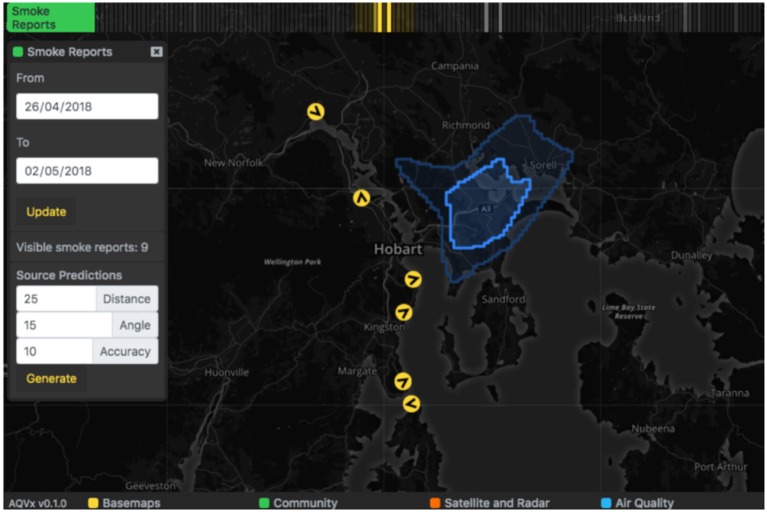
Where multiple smoke reports and orientations are available, a “Source Predictions” function can be used to generate the likely region of smoke source based on the reports visible. The estimated source region is rendered on the map as a blue polygon.

Symptoms and smoke reports are plotted on the map as markers and grouped in hour-long blocks on the relevant time track, and activating one block will display all reports that occurred within that hour on the map (Figure 5. Both types of reports appear as map markers that can be clicked to provide a summary of the report. If available, the photo taken of smoke in the distance can be viewed from the smoke report summary (Figure 6). Markers for smoke reported in the distance display the orientation logged by the device at the time of capture. A “Source Prediction” function displays the likely region of smoke where the orientation and position of multiple smoke reports were available, based on the plume triangulation calculation (Figure 7). The source prediction region appeared as a polygon on the map, surrounding regions where plume triangulation cell values are greater than the median value and indicating likely smoke plume presence.

**Figure 8:**
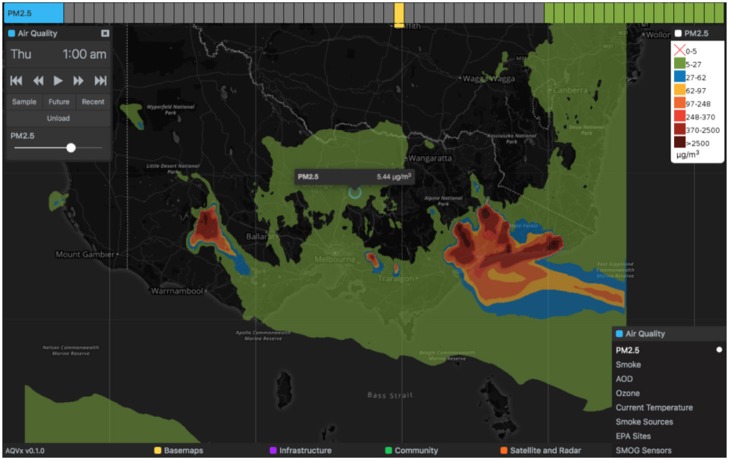
Point values are available for CTM layers by clicking on the map.

Point location pollution concentrations are available for CTM map layers by clicking a point on the map (Figure 8).

## 3. Use Cases

### 3.1. Cross-Referencing CTM PM_2.5_ With Observations

A typical task for an AQVx operator would be to validate the CTM output by cross-referencing with on-the-ground air quality sensors, to confirm similar pollutant concentrations between model and observation, either quantitatively in terms of concentration values at a point, or qualitatively in terms of pollutant plume location and direction of travel. AQVx integrates two sources of on-the-ground air quality sensor data: data from low-cost SMOG sensors, and EPA monitors. An operator first activates the CTM PM_2.5_ data layer and selects the relevant time period from the time track at the top of the app. The “EPA Sites” map layer can then be overlaid, as well as the SMOG sensor map layer, and the operator can hover the mouse pointer over the target time period in any of the tracks to find the corresponding time in the other layers before selecting them for display. Visually the operator would compare the CTM output with the marker colors for the EPA sites and SMOG sensors, or click on the map to view a popup with the local CTM concentration. The marker colors for SMOG sensors and EPA sites are coordinated with the CTM PM_2.5_ layer scale allowing for an easy visual comparison. A video demonstration of this process is available online at: https://vimeo.com/333484388.

### 3.2. Cross-Referencing CTM PM_2.5_ With Fires and Smoke Sources

Where a discrepancy is present between CTM output and other intelligence (e.g., on the-ground sensors or satellite imagery) it is useful to cross-reference current fires and smoke sources (fire incidents and planned burns) as possible sources of inaccuracy in the model, for example fires that may or may not have been included in model generation, due to lack of detection of fire at the time of the model run, or fires that are flagged as continuing after they have been extinguished on the ground. Having loaded the CTM PM_2.5_ layer, the operator is able to overlay the “Himawari Active Fires” and “Geoscience Hotspots” layers, synchronize time series data using the time tracks, and visually compare detected fire presence with the model output, and identify false-positive or negative smoke plume inclusion. A video demonstration of this process is available online at: https://vimeo.com/333485993.

### 3.3. Assessing Community Impacts of Forecast PM_2.5_

Evaluating the potential health impact of forecast PM_2.5_ was a key output of the system. The AQVx operator activates the CTM PM_2.5_ layer and loads the forecast time series. Schools and aged care facility locations are now overlaid as markers. Animation controls are used to easily cycle through the forecast time series, and the operator visually assesses the schools or aged care facilities that fall within regions of elevated PM_2.5_ through the forecast period. For additional information about the schools or aged care facilities that are affected, the operator clicks the icon to view address, phone number and facility details. The operator can also load the NEXIS population density layer to evaluate the impact on densely population areas throughout the forecast time series. A video demonstration of this process is available online at: https://vimeo.com/333485993.

## 4. Discussion

Operators of the AQVx system over the 2019 Victorian prescribed fire season reported that they found the system valuable and useful, particularly in assessing the accuracy and of the AQFx chemical transport model forecasts against on-ground monitoring and field reports. The system was also used by developers of the AQFx model during the catastrophic 2019/2020 wildfire season to compare modeled PM_2.5_ concentration with monitor data and known fire locations, to validate model accuracy and ensure correct paramaterization of active fires in the AQFx model. The need to ensure timely and accurate ingestion of fire location data, including information on when fires are controlled or extinguished, was highlighted by operators, as the AQVx interface enabled the occasional identification of modeled plumes extending from fires that had been extinguished, or the lack of plumes from known fires that had not been incorporated into model inputs. Development of the AQFx model is ongoing, to improve model run time, and to better incorporate new fire ignitions as they occur, through on-demand model runs after fire locations are known, with model chemistry modules disabled to ensure rapid computation.

Symptom reports were submitted by member of the public during the 2019 Victorian prescribed fire season, although user numbers were low during this trial period. The AQVx product will continue to be operational over the 2020 fire season, with additional user recruitment to be encouraged.

The value of geostationary satellite products with sub-hourly imagery (such as Himawari-8) to near-real-time analysis of smoke concentration and dispersion was confirmed by operators, who appreciated the availability of visible animated smoke plumes in the interface, to compare with model output. Work is ongoing to incorporate an aerosol optical depth (AOD) product (28) derived from Himawari-8 into the available data layers. AOD provides a useful proxy for ground-level PM concentration beyond the range of fixed PM monitoring sites, but existing satellite-based AOD products are only available at daily timesteps. Frequent AOD grids from a geostationary platform will greatly assist in chemical transport model validation. There is also ongoing research into the derivation of active fire hotspot locations from the Himawari-8 platform (29, 30), which will provide rapid detection of fire ignition for incorporation into emissions models, as well as live maps of fire spread.

The current tool provides overlays of sensitive populations, both as a population density layer and point locations of schools and aged care facilities. In the state of Victoria, the Environmental Protection Authority (EPA Victoria) is responsible for issuing air quality notices to inform the community about potential smoke impacts, and actions that can be taken to reduce exposure for sensitive groups, such as remaining inside, avoiding exercise, or operating indoor air filters or air conditioners. AQVx enables more precise targeting of these messages by assisting in the identification of potential smoke impacts on a local scale. A potential future extension to AQVx is the incorporation of a health impact assessment tool. Forecast pollutant concentrations, combined with mapped population density and incidence rates for hospitalization and death (31), can be used to predict and quantify significant health impacts from prescribed or wildfire smoke. Real-time health impact assessment would significantly improve fire managers' capacity for planning and understanding prescribed burn impact, and integrating these calculations into AQVx is a promising direction for future research.

Fire management through prescribed burn planning is increasingly under pressure to incorporate smoke management, through modeling and projecting smoke impacts and identifying episodes smoke exposure of sensitive populations. Recent increases in the availability of rapid, high resolution remote sensing data on landscape fire and smoke, along with innovations in pollutant transport modeling and low-cost on-ground sensors, provide fire managers with increased opportunities for understanding and monitoring smoke. AQVx leverages the wide variety of real-time spatial data, satellite imagery and model output now available to provide a simple, functional mapping interface that allows for spatial and temporal overlay of diverse data sources in order to facilitate smoke monitoring and inform transport model validation.

## Data Availability Statement

The AQVx system is currently unavailable for public access due to the display of geolocated personal user data and photographs within the system. Interested parties should contact the authors to arrange access or a demonstration of functionality.

## Ethics Statement

The research was approved by the Tasmanian Health and Medical Human Research Ethics Committee (Reference number: H0015006).

## Author Contributions

GW developed the GIS web mapping server pipeline and directional smoke prediction algorithm, and wrote the manuscript. CL developed the AQVx front-end web display system and wrote the manuscript.

### Conflict of Interest

The authors declare that the research was conducted in the absence of any commercial or financial relationships that could be construed as a potential conflict of interest. The reviewer MH and handling Editor declared their shared affiliation at the time of review.
